# Association of hearing loss with cardiovascular and mortality risk in the general population

**DOI:** 10.1038/s41598-025-27832-x

**Published:** 2025-12-05

**Authors:** Omar Hahad, Julia Döge, Katharina Bahr-Hamm, Jasmin Ghaemi Kerahrodi, Katharina Geschke, Konstantin Kontohow-Beckers, Alexander K. Schuster, Emilio Gianicolo, Karl J. Lackner, Julia Weinmann-Menke, Philipp Lurz, Stavros Konstantinides, Philipp S. Wild, Berit Hackenberg

**Affiliations:** 1 Department of Cardiology – Cardiology I, University Medical Center of the Johannes Gutenberg-University Mainz, Langenbeckstraße 1, 55131 Mainz, Germany; 2 German Center for Cardiovascular Research (DZHK), Partner Site Rhine-Main, Mainz, Germany; 3 Department of Otorhinolaryngology, University Medical Center of the Johannes Gutenberg-University Mainz, Mainz, Germany; 4 Department of Psychosomatic Medicine and Psychotherapy, University Medical Center of the Johannes Gutenberg-University Mainz, Mainz, Germany; 5 Department of Psychiatry and Psychotherapy, University Medical Center of the Johannes Gutenberg-University Mainz, Mainz, Germany; 6 Preventive Cardiology and Preventive Medicine, Department of Cardiology, University Medical Center of the Johannes Gutenberg-University Mainz, Mainz, Germany; 7 Department of Ophthalmology, University Medical Center of the Johannes Gutenberg-University Mainz, Mainz, Germany; 8 Institute of Medical Biostatistics, Epidemiology and Informatics, University Medical Center of the Johannes Gutenberg-University Mainz, Mainz, Germany; 9 Institute of Clinical Chemistry and Laboratory Medicine, University Medical Center of the Johannes Gutenberg-University Mainz, Mainz, Germany; 10 Department of Nephrology, I. Department of Medicine, University Medical Center of the Johannes Gutenberg-University Mainz, Mainz, Germany; 11 Center for Thrombosis and Hemostasis (CTH), University Medical Center of the Johannes Gutenberg-University Mainz, Mainz, Germany; 12 Institute for Molecular Biology, Mainz, Germany

**Keywords:** Hearing loss, Cardiovascular disease, Mortality, Diabetes, Epidemiology, Cardiology, Risk factors

## Abstract

Hearing loss affects over 1.5 billion individuals worldwide and is associated with significant challenges, including social isolation and cognitive decline. Emerging evidence suggests a link between hearing loss and cardiovascular diseases. Comprehensive epidemiological studies exploring these associations remain limited. Using data from the Gutenberg Health Study, a population-based cohort of 15,010 participants aged 35–74 years at baseline, we investigated the relationship between hearing loss, cardiovascular diseases and risk factors, and all-cause mortality. Participants underwent extensive audiometric assessments and clinical evaluations. Logistic regression and Cox proportional hazards models were applied to determine associations between hearing loss, cardiovascular health, and mortality, adjusting for potential confounders. Among the 8886 participants with complete hearing data, 35.1% exhibited some degree of hearing loss. Crude models revealed significant associations between hearing loss and various cardiovascular diseases and risk factors. However, these associations lost significance after adjustments for confounders, except for diabetes (adjusted odds ratio 1.24, 95% confidence interval (CI) 1.01–1.51). Hearing loss coincided with higher mortality in unadjusted analyses (hazard ratio 5.64, 95% CI 4.24–7.49), but this relationship disappeared after full adjustment. Hearing loss may serve as an early marker of systemic vascular dysfunction, particularly in diabetes, rather than an independent predictor of cardiovascular disease or mortality. It needs to be determined whether incorporating auditory health into broader cardiovascular assessments could improve early detection and prevention strategies.

## Introduction

Hearing loss is a significant global health concern, affecting over 1.5 billion people worldwide, with nearly 430 million experiencing disabling hearing impairment, according to the World Health Organization^1^. The prevalence of hearing loss increases with age and poses substantial challenges to individuals’ quality of life, including communication difficulties, social isolation, and cognitive decline^2^. Beyond these well-documented consequences, emerging evidence suggests a potential link between hearing loss and cardiovascular diseases^3–6^, which remain the leading cause of mortality globally^7^.

The cochlea, a highly vascularized organ in the inner ear, relies on a complex network of microvasculature for its function. Damage to this microvasculature due to systemic vascular conditions such as hypertension, diabetes mellitus, or atherosclerosis can impair cochlear function and lead to hearing loss^8^. Conversely, hearing impairment may serve as an early marker of systemic vascular dysfunction or be influenced by shared risk factors such as smoking, obesity, and dyslipidemia^3–6^. These overlapping pathways highlight the necessity of understanding the relationship between auditory and cardiovascular health.

Recent studies have also explored the role of oxidative stress and inflammation as common mechanisms underlying both hearing loss and cardiovascular disease^9,10^. Chronic inflammation and endothelial dysfunction are well-established contributors to cardiovascular pathology and may similarly affect cochlear health^11,12^. Furthermore, social isolation resulting from hearing loss has been associated with increased stress and depression, which are recognized risk factors for adverse cardiovascular outcomes^13,14^.

Interestingly, a recent study investigated the combined effect of cardiovascular diseases and hearing loss on all-cause and cardiovascular mortality based on data from 10,614 participants in the National Health and Nutrition Examination Survey (NHANES)^15^. Compared to those without either condition, the risk of all-cause mortality was 1.88 times higher for the cardiovascular disease+/hearing loss- group and 2.19 times higher for the cardiovascular disease+/hearing loss+group. For cardiovascular mortality, risks were 3.66 times higher for cardiovascular disease+/hearing loss- and 2.91 times higher for cardiovascular disease+/hearing loss+. However, no significant increase in mortality was observed for the hearing loss-only group. In contrast, a large prospective study of 13,880 adults from China found no significant association between greater high-frequency hearing loss and the risk of incident coronary heart disease or overall cardiovascular disease after multivariable adjustment^16^.

Taken together, evidence from large-scale epidemiological studies investigating the associations between hearing loss, cardiovascular disease, and related risk factors remains limited and inconclusive. The Gutenberg Health Study (GHS), a population-based cohort with a wide age range, offers a unique platform to examine these relationships in detail. By incorporating comprehensive audiometric assessments and extensive cardiovascular evaluations, the present study seeks to contribute to the understanding of potential associations between hearing loss, cardiovascular diseases, and their shared risk factors, providing insights that may contribute to future research and public health strategies.

## Methods

### The Gutenberg health study—study design and sample

**Fig. 1:**
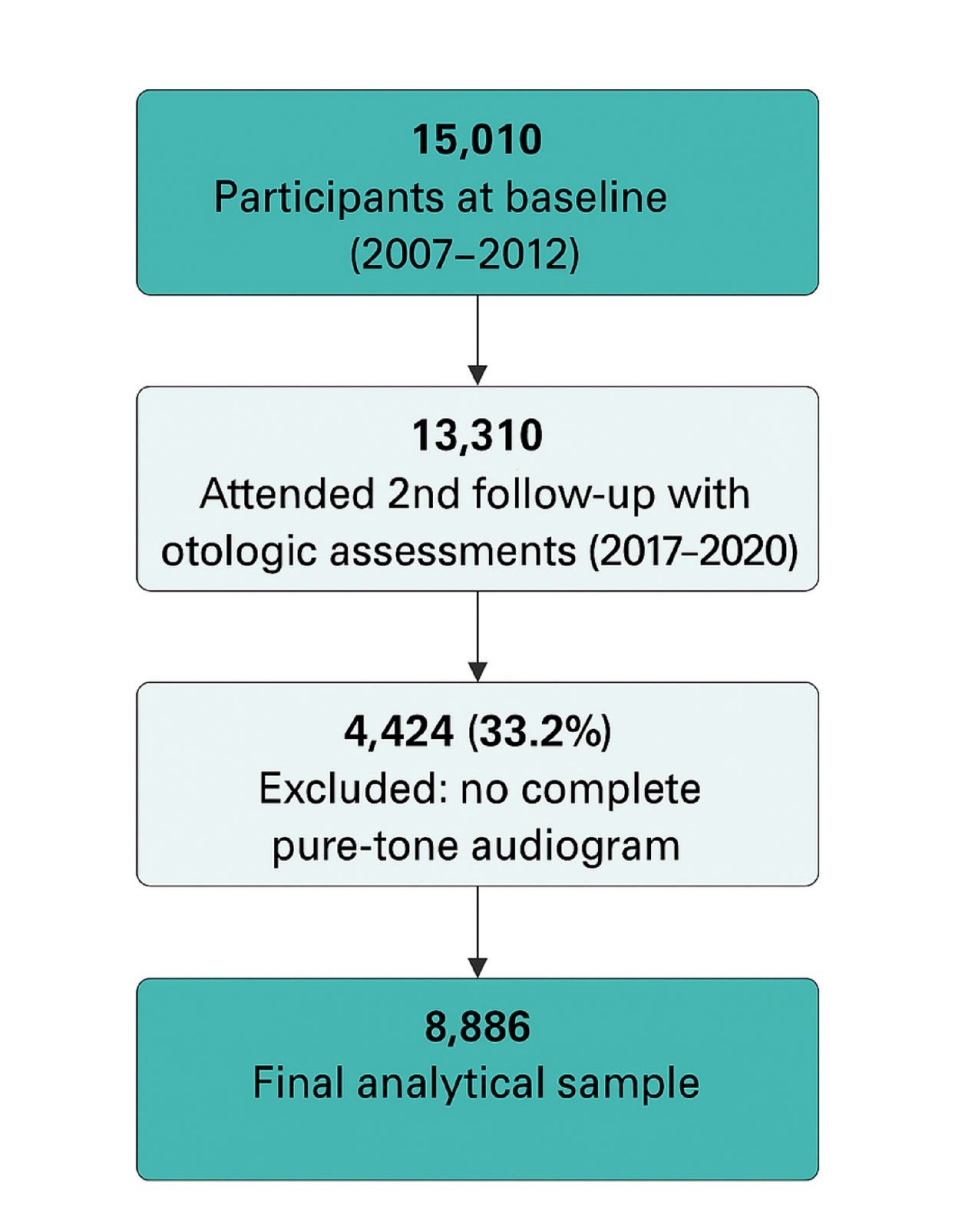
Flow diagram of participant inclusion and exclusion for the Gutenberg Health Study analysis. From 15,010 participants enrolled at baseline (2007–2012), 13,310 attended the second follow-up examination (2017–2020) including otologic assessments. Of these, 4424 participants (33.2%) were excluded due to incomplete pure-tone audiograms, resulting in a final analytical sample of 8,886 participants with complete audiometric data.

We used data from the GHS, a large-scale population-based study comprising 15,010 participants aged 35 to 74 years, referred to as the core cohort. Between 2007 and 2012, these individuals underwent an extensive baseline examination lasting five hours, conducted at the University Medical Center Mainz, Germany^17–19^. The assessments were carried out using standardized protocols and included a range of clinical tests and structured interviews. Follow-up evaluations took place in two phases: from 2012 to 2017 (5 years post-baseline) and from 2017 to 2022 (10 years post-baseline). During the second follow-up period (2017 to 2020), the study expanded to include otologic assessments^20^ (Fig. 1). Ethical approval for the GHS was granted by the ethics committee of the Statutory Physician Board of the State Rhineland-Palatinate (approval number 837.020.07(5555)), ensuring compliance with the ethical principles of the Declaration of Helsinki. Informed written consent was obtained from all participants prior to their inclusion in the study.

### Hearing loss

Frequent otological symptoms were evaluated using computer-assisted personal interviews as part of a comprehensive testing protocol^21^. These interviews provided essential information on participants hearing health prior to otoscopy and the initiation of pure-tone audiometry. Pure-tone audiometry was performed separately for each ear to measure both air and bone conduction thresholds at the following frequencies: 0.125 kHz, 0.25 kHz, 0.5 kHz, 0.75 kHz, 1 kHz, 2 kHz, 3 kHz, 4 kHz, 6 kHz, 8 kHz, and 10 kHz. All tests were conducted in a soundproof booth using the Auritec® AT1000 clinical audiometer to ensure accurate and reliable results.

Hearing loss was classified based on the average audiometric thresholds measured at 0.5, 1, 2, and 4 kHz in the better ear according to the classification of the World Health Organization. The categories included no (< 20 dB), mild (20–34.9 dB), moderate (35–49.9 dB), moderately severe (50–64.9 dB), severe (65–79.9 dB), profound (80–94.9 dB), and complete (95 dB or greater in the better ear) hearing loss^21^.

### Cardiovascular disease, risk factors, and mortality

Cardiovascular disease prevalence was assessed by reviewing medical records that included diagnoses from physicians and those made during study visits. The definition encompassed conditions such as coronary artery disease, myocardial infarction, atrial fibrillation, chronic heart failure, stroke, and peripheral artery disease. Mortality updates were obtained quarterly through inquiries with registry offices and the Rhineland-Palatinate mortality registry, with official death certificates reviewed for accuracy. Socioeconomic status was measured using a validated index incorporating education, occupation, and income, with scores ranging from 3 (lowest) to 21 (highest), where higher scores reflected higher status^22^. Smoking status categorized participants as either current or non-smokers. Current smokers were defined as individuals smoking at least one cigarette daily, seven weekly, or one pack monthly for six consecutive months. Non-smokers included those who had never smoked, former smokers abstinent for over six months, and occasional smokers. Diabetes mellitus was identified based on physician diagnosis, antidiabetic medication use, fasting glucose ≥ 126 mg/dL after overnight fasting, non-fasting glucose ≥ 200 mg/dL, or HbA1c ≥ 6.5%. Arterial hypertension was defined as the use of antihypertensive medication or average resting systolic blood pressure ≥ 140 mmHg or diastolic blood pressure ≥ 90 mmHg (calculated from the second and third readings after rest periods of 8 and 11 min). Obesity was classified as a body mass index of 30 kg/m^2^ or higher^23^. Dyslipidemia was determined by a physician’s diagnosis, a LDL to HDL cholesterol ratio exceeding 3.5, or triglycerides ≥ 150 mg/dL. Family history of myocardial infarction or stroke was noted for first-degree relatives who had experienced such events, defined as females aged ≤ 65 years or males aged ≤ 60 years.

### Statistical analysis

Participants with incomplete data on hearing loss were excluded from the analysis. The characteristics of the study sample are presented as follows: continuous variables are reported as mean and standard deviation. In cases where skewness exceeded 1, they are summarized as median with interquartile range (Q1, Q3). Discrete variables are described using absolute and relative frequencies. For comparison between groups, *p* values for continuous variables were calculated using the t test, while for dichotomous variables the Chi-squared test was applied. Binary logistic regression analysis was conducted to determine the association between hearing loss as well hearing threshold levels (independent variables) and prevalent cardiovascular disease and cardiovascular risk factors (dependent variables) with odds ratios (OR), 95% confidence intervals (CI), and *p* values reported. Furthermore, hazard ratios (HR) and their corresponding 95% CI were determined using Cox regression models to examine the association between hearing loss as well hearing threshold levels (independent variables) and the risk of all-cause mortality (dependent variable). Sequential adjustments were made in the models: Model 1 was crude. Model 2 was additionally adjusted for sex, age, and socioeconomic status. Model 3 was additionally adjusted for current smoking, diabetes mellitus, arterial hypertension, obesity, dyslipidemia, and family history of myocardial infarction or stroke. *P* values were interpreted as continuous measures of evidence against the null hypothesis and were therefore reported precisely. Statistical analyses were conducted using the R software package (http://www.r-project.org/).

## Results

### Study sample characteristics

Among 8,886 participants with complete hearing data, 3,123 (35.1%) had some degree of hearing loss (Table 1). Participants with hearing loss were older and had lower socioeconomic status compared to those with normal hearing (*p* < 0.0001). Hearing loss also coincided with a higher prevalence of cardiovascular risk factors, including arterial hypertension, diabetes mellitus, obesity, dyslipidemia, and family history of myocardial infarction or stroke. For instance, the prevalence of arterial hypertension increased from 37.8% in individuals with normal hearing to 68.7% in those with hearing loss (*p* < 0.0001).

**Table 1:** Characteristics of the Gutenberg Health Study sample stratified by the presence of hearing loss (*N* = 8,886).

	No hearing loss (n = 5,763)	Hearing loss (n = 3,123)	*P* value
Sociodemographic			
Women—% (no.)	52.1 (3,000)	44.1 (1,377)	**< 0.0001**
Age (years)—mean ± SD	51.4 ± 11.9	69.6 ± 10.2	**< 0.0001**
Socioeconomic status—mean ± SD	15.29 ± 3.95	13.07 ± 4.23	**< 0.0001**
Cardiovascular risk factors—% (no.)			
Current smoking	15.1 (868)	11.3 (345)	**< 0.0001**
Diabetes mellitus	5.2 (298)	15.4 (480)	**< 0.0001**
Arterial hypertension	37.8 (2,177)	68.7 (2,144)	**< 0.0001**
Obesity	23.3 (1,340)	28.8 (898)	**< 0.0001**
Dyslipidemia	21.5 (1,238)	40.5 (1,262)	**< 0.0001**
Family history of myocardial infarction or stroke	1.9 (110)	3.2 (101)	**0.00014**
Cardiovascular disease—% (no.)			
Myocardial infarction	0.5 (28)	2.4 (73)	**< 0.0001**
Stroke	0.5 (30)	2.5 (77)	**< 0.0001**
Atrial fibrillation	1.0 (60)	5.4 (168)	**< 0.0001**
Peripheral artery disease	1.5 (84)	4.0 (121)	**< 0.0001**
Coronary artery disease	1.2 (67)	4.3 (131)	**< 0.0001**
Chronic heart failure	1.1 (62)	3.2 (99)	**< 0.0001**
Any cardiovascular disease	4.8 (274)	16.7 (516)	**< 0.0001**
Hearing threshold levels (dB) at specific frequencies ((k)Hz)			
Left ear			
500 Hz—median (Q1/Q3)	10.00 (5.00/15.00)	25.00 (15.00/30.00)	**< 0.0001**
1 kHz—median (Q1/Q3)	10.00 (5.00/11.00)	25.00 (15.00/31.00)	**< 0.0001**
2 kHz—median (Q1/Q3)	10.00 (5.00/15.00)	35.00 (25.00/45.00)	**< 0.0001**
4 kHz—mean ± SD	19.15 ± 12.63	51.37 ± 17.10	**< 0.0001**
Right ear			
500 Hz—median (Q1/Q3)	10.00 (6.00/15.00)	25.00 (15.00/31.00)	**< 0.0001**
1 kHz—median (Q1/Q3)	10.00 (5.00/15.00)	25.00 (15.00/34.00)	**< 0.0001**
2 kHz—median (Q1/Q3)	10.00 (5.00/15.00)	30.00 (25.00/45.00)	**< 0.0001**
4 kHz—mean ± SD	18.11 ± 11.97	49.97 ± 17.81	**< 0.0001**
Hearing threshold level (dB)			
Audiometric value (dB)—median (Q1/Q3)	10.50 (7.50/14.25)	28.75 (23.75/36.96)	**< 0.0001**
Degree of hearing loss—% (no.)			
No	100 (5,763)	0 (0)	–
Mild	–	69.3 (2,164)	–
Moderate	–	24.4 (763)	–
Moderately severe	–	5.5 (173)	–
Severe	–	0.6 (18)	–
Profound	–	0.2 (5)	–
Complete	–	0 (0)	–

Continuous variables are shown as mean and standard deviation or if skewness > 1 by median (Q1, Q3). Discrete variables are described as relative and absolute frequencies. *P* values for comparison between groups are estimated using the t test for continuous variables and the Chi-squared test for dichotomous variables.

Socioeconomic status score ranges from 3 to 21 with higher values indicating higher status.

Significant values are in bold.

### Hearing loss and cardiovascular disease

In the crude model (Model 1), individuals with hearing loss showed higher odds for various cardiovascular diseases, including myocardial infarction, stroke, atrial fibrillation, peripheral artery disease, coronary artery disease, chronic heart failure, and any cardiovascular disease (*p* < 0.0001, Tables 2 and 3). However, after adjusting for age, sex, socioeconomic status (Model 2), and additional cardiovascular risk factors (Model 3), these associations lost statistical significance.

**Table 2:** Odds ratios and 95% confidence intervals were calculated using binary logistic regression models to examine the association between hearing loss (independent variable, no vs. hearing loss) and prevalent cardiovascular disease (dependent variable) in the Gutenberg Health Study.

	*N*/events	Model 1		Model 2		Model 3	
		OR [95% CI]	*P* value	OR [95% CI]	*P* value	OR [95% CI]	*P* value
Myocardial infarction	8,473/92	4.939 [3.187; 7.652]	**< 0.0001**	1.557 [0.892; 2.716]	0.12	1.620 [0.921; 2.849]	0.094
Stroke	8,475/94	4.849 [3.173; 7.411]	**< 0.0001**	0.908 [0.541; 1.526]	0.72	0.880 [0.513; 1.511]	0.64
Atrial fibrillation	8,466/209	5.440 [4.037; 7.329]	**< 0.0001**	1.253 [0.870; 1.805]	0.23	1.243 [0.857; 1.803]	0.25
Peripheral artery disease	8,397/182	2.773 [2.092; 3.676]	**< 0.0001**	1.172 [0.809; 1.698]	0.40	1.052 [0.722; 1.534]	0.79
Coronary artery disease	8,407/182	3.802 [2.823; 5.120]	**< 0.0001**	1.128 [0.773; 1.645]	0.53	1.144 [0.774; 1.691]	0.50
Chronic heart failure	8,392/148	3.058 [2.220; 4.214]	**< 0.0001**	1.316 [0.869; 1.993]	0.19	1.292 [0.848; 1.970]	0.23
Any cardiovascular disease	8,443/725	4.019 [3.446; 4.687]	**< 0.0001**	1.188 [0.975; 1.447]	0.087	1.154 [0.942; 1.415]	0.17

*N*/events denotes model 3.

Model 1 was crude.

Model 2 was additionally adjusted for sex, age, and socioeconomic status.

Model 3 was additionally adjusted for current smoking, diabetes mellitus, arterial hypertension, obesity, dyslipidemia, and family history of myocardial infarction or stroke.

Significant values are in bold.

**Table 3:** Odds ratios and 95% confidence intervals were calculated using binary logistic regression models to examine the association between hearing threshold levels (independent variable, per dB increase) and prevalent cardiovascular disease (dependent variable) in the Gutenberg Health Study.

	*N*/events	Model 1		Model 2		Model 3	
		OR per point increase [95% CI]	*P* value	OR per point increase [95% CI]	*P* value	OR per point increase [95% CI]	*P* value
Myocardial infarction	8,473/92	1.043 [1.031; 1.056]	**< 0.0001**	0.995 [0.976; 1.014]	0.60	0.996 [0.976; 1.016]	0.68
Stroke	8,475/94	1.060 [1.048; 1.072]	**< 0.0001**	1.013 [0.996; 1.030]	0.14	1.015 [0.996; 1.034]	0.11
Atrial fibrillation	8,466/209	1.052 [1.043; 1.061]	**< 0.0001**	0.998 [0.986; 1.011]	0.78	1.00 [0.987; 1.013]	1.00
Peripheral artery disease	8,397/182	1.035 [1.025; 1.044]	**< 0.0001**	1.001 [0.988; 1.015]	0.84	0.994 [0.979; 1.009]	0.42
Coronary artery disease	8,407/182	1.042 [1.033; 1.052]	**< 0.0001**	0.996 [0.983; 1.010]	0.59	0.998 [0.983; 1.013]	0.76
Chronic heart failure	8,392/148	1.036 [1.025; 1.046]	**< 0.0001**	1.003 [0.988; 1.018]	0.72	1.003 [0.988; 1.019]	0.68
Any cardiovascular disease	8,443/725	1.050 [1.045; 1.055]	**< 0.0001**	1.003 [0.995; 1.010]	0.47	1.002 [0.994; 1.010]	0.67

*N*/events denotes model 3.

Model 1 was crude.

Model 2 was additionally adjusted for sex, age, and socioeconomic status.

Model 3 was additionally adjusted for current smoking, diabetes mellitus, arterial hypertension, obesity, dyslipidemia, and family history of myocardial infarction or stroke.

Significant values are in bold.

### Hearing loss and cardiovascular risk factors

In the crude model (Model 1), individuals with hearing loss had higher odds of arterial hypertension, dyslipidemia, obesity, and diabetes mellitus (*p* < 0.0001, Table 4). After comprehensive adjustment, these associations disappeared, except for diabetes mellitus, which remained robust (OR 1.24, 95% CI 1.01–1.51).

**Table 4:** Odds ratios and 95% confidence intervals were calculated using binary logistic regression models to examine the association between hearing loss (independent variable, no vs. hearing loss) and prevalent cardiovascular risk factors (dependent variable) in the Gutenberg Health Study.

	*N*/events	Model 1		Model 2		Model 3	
		OR [95% CI]	*P* value	OR [95% CI]	*P* value	OR [95% CI]	*P* value
Arterial hypertension	8,495/4,116	3.603 [3.285; 3.952]	**< 0.0001**	1.078 [0.954; 1.219]	0.23	1.085 [0.955; 1.234]	0.21
Dyslipidemia	8,495/2,374	2.475 [2.251; 2.723]	**< 0.0001**	0.979 [0.863; 1.111]	0.74	0.935 [0.822; 1.064]	0.31
Obesity	8,495/2,122	1.333 [1.208; 1.471]	**< 0.0001**	0.952 [0.836; 1.084]	0.46	0.917 [0.800; 1.050]	0.21
Diabetes mellitus	8,495/727	3.333 [2.863; 3.879]	**< 0.0001**	1.266 [1.039; 1.542]	**0.019**	1.238 [1.013; 1.514]	**0.037**

*N*/events denotes model 3.

Model 1 was crude.

Model 2 was additionally adjusted for sex, age, and socioeconomic status.

Model 3 was additionally adjusted for current smoking, diabetes mellitus, arterial hypertension, obesity, dyslipidemia, and family history of myocardial infarction or stroke (except for the specific variable that constituted the dependent variable in this context).

Significant values are in bold.

Similarly, each 1 dB increase in hearing threshold levels was associated with higher odds of cardiovascular risk factors in the crude model, while only the relationship with diabetes mellitus remained robust (OR 1.01, 95% CI 1.00–1.02, Table 5).

**Table 5:** Odds ratios and 95% confidence intervals were calculated using binary logistic regression models to examine the association between hearing threshold levels (independent variable, per dB increase) and prevalent cardiovascular risk factors (dependent variable) in the Gutenberg Health Study.

	*N*/events	Model 1		Model 2		Model 3	
		OR per point increase [95% CI]	*P* value	OR per point increase [95% CI]	*P* value	OR per point increase [95% CI]	*P* value
Arterial hypertension	8,495/4,116	1.063 [1.059; 1.068]	**< 0.0001**	1.003 [0.998; 1.009]	0.22	1.002 [0.996; 1.008]	0.51
Dyslipidemia	8,495/2,374	1.040 [1.036; 1.044]	**< 0.0001**	1.001 [0.996; 1.006]	0.77	0.998 [0.993; 1.004]	0.59
Obesity	8,495/2,122	1.015 [1.011; 1.019]	**< 0.0001**	1.004 [0.999; 1.010]	0.10	1.002 [0.996; 1.007]	0.55
Diabetes mellitus	8,495/727	1.047 [1.041; 1.052]	**< 0.0001**	1.012 [1.005; 1.020]	**0.0013**	1.011 [1.003; 1.018]	**0.0082**

*N*/events denotes model 3.

Model 1 was crude.

Model 2 was additionally adjusted for sex, age, and socioeconomic status.

Model 3 was additionally adjusted for current smoking, diabetes mellitus, arterial hypertension, obesity, dyslipidemia, and family history of myocardial infarction or stroke (except for the specific variable that constituted the dependent variable in this context).

Significant values are in bold.

### Hearing loss and all-cause mortality

**Fig. 2:**
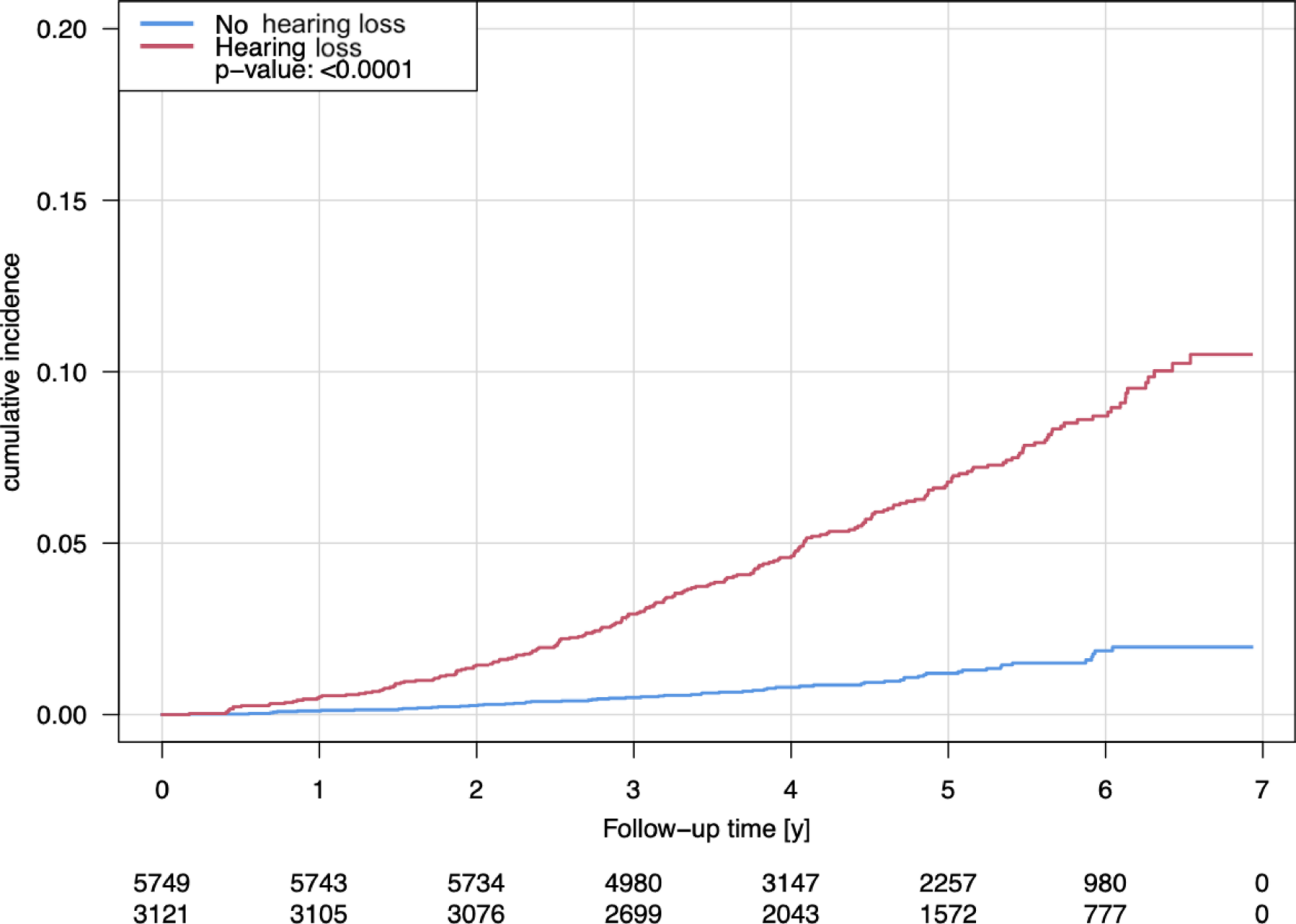
Kaplan–Meier curves illustrating the association between the presence of hearing loss and risk of all-cause mortality in the Gutenberg Health Study. The *P* value corresponds to the log-rank test.

Over a follow-up period of 6.93 years, a total of 273 deaths were recorded, with 62 occurring in individuals without hearing loss and 211 in individuals with hearing loss. In crude analyses, hearing loss was associated with increased all-cause mortality (HR 5.64, 95% CI 4.24–7.49, Table 6 and Fig. 2). After adjusting for potential confounders (Model 3), the association was no longer statistically significant. Similarly, increasing hearing threshold levels were associated with higher all-cause mortality risk in unadjusted analyses, but this association disappeared after full adjustment.

**Table 6:** Hazard ratios (HR) and 95% confidence intervals were calculated using Cox regression models to examine the association between hearing loss or hearing threshold levels (independent variables) and risk of all-cause mortality (dependent variable) in the Gutenberg Health Study.

All-cause mortality	*N*/event*s*	Model 1		Model 2		Model 3	
		HR [95% CI]	*P* value	HR [95% CI]	*P* value	HR [95% CI]	*P* value
Hearing loss (no vs. hearing loss)	8,478/255	5.636 [4.244; 7.485]	**< 0.0001**	1.075 [0.775; 1.491]	0.67	1.006 [0.722; 1.401]	0.97
Hearing threshold levels (per dB increase)	8,478/255	1.058 [1.051; 1.065]	**< 0.0001**	1.010 [1.000; 1.021]	0.051	1.007 [0.996; 1.018]	0.19

*N* denotes model 3.

Model 1 was crude.

Model 2 was additionally adjusted for sex, age, and socioeconomic status.

Model 3 was additionally adjusted for current smoking, diabetes mellitus, arterial hypertension, obesity, dyslipidemia, and family history of myocardial infarction or stroke.

Significant values are in bold.

## Discussion

The present study, using data from the GHS, provides valuable insights into the relationship between hearing loss, cardiovascular disease, and mortality risk in the general population. Our findings contribute to the growing body of evidence associating auditory function with cardiovascular health and all-cause mortality. Our analysis revealed that individuals with hearing loss had higher odds of various cardiovascular diseases and risk factors in crude models. However, after comprehensive adjustment for potential confounders, most of these associations lost significance. Notably, the relationship between hearing loss and diabetes mellitus remained even after full adjustment.

Our crude findings are consistent with prior studies reporting positive associations between hearing loss and cardiovascular disease, whereas our fully adjusted results contrast with these studies that still observed significant effects after multivariable adjustment^6,24–27^. This discrepancy likely reflects differences in study design and exposure assessment, such as the use of self-reported hearing measures, varying definitions of cardiovascular outcomes, and differences in population characteristics, including age structure, ethnicity, and underlying risk profiles. In addition, inconsistencies in the extent of confounder adjustment, residual confounding, and potential over adjustment for mediating factors such as hypertension may further contribute to the divergent findings across studies. However, the attenuation of these associations after adjusting for age, sex, and socioeconomic status, with no substantial changes following additional adjustment for cardiovascular risk factors, suggests that the observed relationships in our sample may be largely explained by the confounding effects of age, sex, and socioeconomic status rather than direct causal pathways.

In contrast, the persistent association between hearing loss and diabetes mellitus, even after comprehensive adjustment, supports the hypothesis that hearing loss may serve as an early marker of systemic vascular dysfunction. Previous studies have attributed this relationship to the detrimental effects of diabetes on the microvasculature of the inner ear, where impaired microvascular function could compromise cochlear health^28–30^. This notion aligns with findings from the GHS, where we previously demonstrated that microvascular endothelial dysfunction, assessed by digital volume plethysmography, predicted the onset of prediabetes and diabetes mellitus^17^. These results suggest that hearing loss may reflect early systemic vascular changes associated with diabetes, reinforcing the importance of considering auditory function as part of broader cardiovascular and metabolic health assessments. However, from an ageing perspective, this association should be interpreted with caution^31^. Age-related metabolic and vascular changes may contribute to both impaired hearing and glucose dysregulation, potentially explaining part of their co-occurrence. In addition, survival and selection bias related to older age cannot be fully excluded, as individuals with advanced age and severe comorbidities are less likely to participate or remain in the cohort. These aspects should be considered when interpreting the observed association between hearing loss and diabetes. The attenuation of associations between hearing loss and other cardiovascular risk factors after adjustment highlights the complexity of these relationships, which are likely influenced by a combination of vascular, metabolic, and demographic factors.

Our study also highlights the complexity of mortality risk assessment in individuals with hearing loss. While the initial association between hearing impairment and increased all-cause mortality was significant, the attenuation of this relationship after comprehensive adjustment suggests that hearing loss may be a marker of overall health status or sign of aging rather than an independent risk factor for mortality. However, previous systematic reviews and meta-analyses have shown a significant association between hearing loss and increased all-cause mortality, even after accounting for demographics and comorbidities. Additionally, a dose–response relationship has been identified, with mortality risk doubling for every 30 dB increase in hearing loss severity^32,33^.

Our study has several strengths, including its large, population-based cohort with comprehensive health data, standardized protocols for hearing assessment and cardiovascular evaluation, and longitudinal design allowing for mortality follow-up. However, we acknowledge certain limitations, such as the cross-sectional nature of the hearing loss and cardiovascular disease data, which limits causal inferences. Additionally, there is potential for residual confounding despite comprehensive adjustment, and the generalizability of our findings to populations outside of the study region may be limited. While our findings do not support an independent association between hearing loss and cardiovascular outcomes and mortality after adjustment, it remains to be determined whether incorporating hearing health into overall cardiovascular risk assessment provides significant clinical value. The persistent association with diabetes mellitus suggests that individuals with hearing loss may benefit from targeted screening for metabolic disorders. Future research should focus on longitudinal studies to elucidate the temporal relationship between hearing loss and cardiovascular outcomes. Furthermore, data on environmental and occupational noise exposure were not available for the present analysis. As noise exposure is a causal upstream factor for hearing loss and also contribute to cardiovascular risk, this limitation precluded further exploration of its mediating or confounding role. Future analyses incorporating modeled environmental and occupational noise data will be valuable to disentangle these interrelationships. Generally, investigation of potential shared pathophysiological mechanisms underlying both hearing loss and cardiovascular disease is warranted. Additionally, evaluation of whether interventions for hearing loss can impact cardiovascular risk and overall mortality would provide valuable insights for clinical practice and public health strategies.

## Data Availability

The analysis presents clinical data of a large-scale population-based cohort with ongoing follow-up examinations. This project constitutes a major scientific effort with high methodological standards and detailed guidelines for analysis and publication to ensure scientific analyses on the highest level. Therefore, data are not made available for the scientific community outside the established and controlled workflows and algorithms. To meet the general idea of verification and reproducibility of scientific findings, we offer access to data at the local database in accordance with the ethics vote on request at any time. The GHS steering committee, which comprises a member of each involved department and the head of the GHS, convenes once a month. The steering committee decides on internal and external access of researchers and use of the data and biomaterials based on a research proposal to be supplied by the researcher. Interested researchers make their requests to the head of the GHS (Philipp S. Wild, philipp.wild@unimedizin-mainz.de).

## References

[1] Deafness and hearing loss: World Health Organization. Updated 2024. Available from: https://www.who.int/news-room/fact-sheets/detail/deafness-and-hearing-loss.

[2] Collaborators GBDHL. Hearing loss prevalence and years lived with disability, 1990–2019: Findings from the Global Burden of Disease Study 2019. *Lancet*. **397**(10278), 996–1009 (2021). 10.1016/S0140-6736(21)00516-X PMC7960691 33714390

[3] Baiduc, R. R., Sun, J. W., Berry, C. M., Anderson, M. & Vance, E. A. Relationship of cardiovascular disease risk and hearing loss in a clinical population. *Sci. Rep.* **13**(1), 1642 (2023). 36717643 10.1038/s41598-023-28599-9 PMC9886989

[4] Wattamwar, K. et al. Association of cardiovascular comorbidities with hearing loss in the older old. *JAMA Otolaryngol. Head Neck Surg.* **144**(7), 623–629 (2018). 29902313 10.1001/jamaoto.2018.0643 PMC6145783

[5] Mick, P. T. et al. Associations between cardiovascular risk factors and audiometric hearing: Findings from the Canadian longitudinal study on aging. *Ear. Hear.* **44**(6), 1332–1343 (2023). 37122082 10.1097/AUD.0000000000001370 PMC10583941

[6] Tan, C. J. et al. Association between hearing loss and cardiovascular disease: A meta-analysis. *Otolaryngol. Head. Neck. Surg.* **170**(3), 694–707 (2024). 38063267 10.1002/ohn.599

[7] Cardiovascular diseases (CVDs): World Health Organization (2021). Available from: https://www.who.int/health-topics/cardiovascular-diseases#tab=tab_1.

[8] Neng, L. & Shi, X. Vascular pathology and hearing disorders. *Curr. Opin. Physiol.* **18**, 79–84 (2020).

[9] Li, P. et al. Mitochondrial dysfunction in hearing loss: Oxidative stress, autophagy and NLRP3 inflammasome. *Front. Cell Dev. Biol.* **11**, 1119773 (2023). 36891515 10.3389/fcell.2023.1119773 PMC9986271

[10] Maniaci, A. et al. Hearing loss and oxidative stress: A comprehensive review. *Antioxidants (Basel).* **13**(7), 842 (2024). 39061910 10.3390/antiox13070842 PMC11274311

[11] Cavallaro, G. et al. Endothelial dysfunction and metabolic disorders in patients with sudden sensorineural hearing loss. *Medicina (Kaunas).* **59**(10), 1718 (2023). 37893435 10.3390/medicina59101718 PMC10608295

[12] Hou, Y. & Liu, B. The role of vascular endothelial dysfunction in hypertension with hearing loss. *Angiology.*10.1177/00033197241247076 (2024). 38626404 10.1177/00033197241247076

[13] Kim, S. Y., Min, C., Lee, C. H., Park, B. & Choi, H. G. Bidirectional relation between depression and sudden sensorineural hearing loss: Two longitudinal follow-up studies using a national sample cohort. *Sci. Rep.* **10**(1), 1482 (2020). 32001781 10.1038/s41598-020-58547-w PMC6992784

[14] Cormier, K., Brennan, C. & Sharma, A. Hearing loss and psychosocial outcomes: Influences of social emotional aspects and personality. *PLoS ONE* **19**(6), e0304428 (2024). 38865302 10.1371/journal.pone.0304428 PMC11168651

[15] Li, L., Li, L., Qin, C., et al. Additive impact of cardiovascular diseases and hearing loss on all-cause and cardiovascular mortality: A longitudinal nationwide population-based study, 02 December 2024, PREPRINT (Version 1) available at Research Square (2024). 10.21203/rs.3.rs-5301331/v1.

[16] Yang, L. et al. Hearing loss is associated with increased risk of incident stroke but not coronary heart disease among middle-aged and older Chinese adults: The Dongfeng-Tongji cohort study. *Environ Sci Pollut Res Int.* **29**(14), 21198–21209 (2022). 34755295 10.1007/s11356-021-17324-6

[17] Hahad, O. et al. Endothelial function assessed by digital volume plethysmography predicts the development and progression of type 2 diabetes mellitus. *J. Am. Heart Assoc.* **8**(20), e012509 (2019). 31583936 10.1161/JAHA.119.012509 PMC6818038

[18] Hahad, O. et al. Cigarette smoking is related to endothelial dysfunction of resistance, but not conduit arteries in the general population-results from the Gutenberg health study. *Front. Cardiovasc. Med.* **8**, 674622 (2021). 34095261 10.3389/fcvm.2021.674622 PMC8169997

[19] Wild, P. S. et al. The Gutenberg health study. *Bundesgesundheitsblatt Gesundheitsforschung Gesundheitsschutz.* **55**(6–7), 824–829 (2012). 22736163 10.1007/s00103-012-1502-7

[20] Hackenberg, B. et al. Tinnitus and its relation to depression, anxiety, and stress-a population-based cohort study. *J. Clin. Med.* **12**(3), 1169 (2023). 36769823 10.3390/jcm12031169 PMC9917824

[21] Doge, J. et al. The prevalence of hearing loss and provision with hearing aids in the Gutenberg health study. *Dtsch. Arztebl. Int.* **120**, 99–106 (2023). 36519221 10.3238/arztebl.m2022.0385 PMC10132285

[22] Lampert, T., Kroll, L. E., Muters, S. & Stolzenberg, H. Measurement of the socioeconomic status within the German Health Update 2009 (GEDA). *Bundesgesundheitsblatt Gesundheitsforschung Gesundheitsschutz* **56**(1), 131–143 (2013). 23275948 10.1007/s00103-012-1583-3

[23] Obesity: preventing and managing the global epidemic. Report of a WHO consultation. *World Health Organ. Tech. Rep. Ser*. **894**, 1–253 (2000). 11234459

[24] He, J., Tang, X., Jiang, M. & Zheng, X. Associations between vision and hearing impairment and cardiovascular diseases: A longitudinal cohort of middle-aged and older adults in China. *J. Am. Heart Assoc.* **13**(21), e034851 (2024). 39435714 10.1161/JAHA.124.034851 PMC11935677

[25] Fang, Q. et al. Hearing loss is associated with increased CHD risk and unfavorable CHD-related biomarkers in the Dongfeng-Tongji cohort. *Atherosclerosis* **271**, 70–76 (2018). 29477559 10.1016/j.atherosclerosis.2018.01.048

[26] Tan, H. E. et al. Associations between cardiovascular disease and its risk factors with hearing loss-A cross-sectional analysis. *Clin. Otolaryngol.* **43**(1), 172–181 (2018). 28703883 10.1111/coa.12936

[27] Baiduc, R. R. et al. Hearing loss and cardiovascular disease risk profiles: Data from the Hispanic community health study/study of Latinos. *J. Am. Acad. Audiol.* **33**(9/10), 445–459 (2022). 39271108 10.1055/s-0042-1758529

[28] Smith, T. L., Raynor, E., Prazma, J., Buenting, J. E. & Pillsbury, H. C. Insulin-dependent diabetic microangiopathy in the inner ear. *Laryngoscope.* **105**(3 Pt 1), 236–240 (1995). 7877409 10.1288/00005537-199503000-00002

[29] Fukushima, H. et al. Effects of type 2 diabetes mellitus on cochlear structure in humans. *Arch. Otolaryngol. Head Neck Surg.* **132**(9), 934–938 (2006). 16982969 10.1001/archotol.132.9.934

[30] Felicio, J. S. et al. Cochlear dysfunction and microvascular complications in patients with type 1 diabetes mellitus. *Diabetol. Metab. Syndr.* **10**, 81 (2018). 30455746 10.1186/s13098-018-0380-z PMC6230237

[31] He, Y., Karhunen, V., Pulakka, A., Kantomaa, M. & Sebert, S. A bidirectional Mendelian randomisation study to evaluate the relationship between body constitution and hearing loss. *Sci**. Rep.. Rep.* **13**(1), 18434 (2023). 37891192 10.1038/s41598-023-44735-x PMC10611773

[32] Hsu, A. K. et al. Associations among hearing loss, hospitalization, readmission and mortality in older adults: A systematic review. *Geriatr. Nurs.* **40**(4), 367–379 (2019). 30851994 10.1016/j.gerinurse.2018.12.013

[33] Tan, B. K. J. et al. Associations of hearing loss and dual sensory loss with mortality: A systematic review, meta-analysis, and meta-regression of 26 observational studies with 1 213 756 participants. *JAMA Otolaryngol. Head Neck Surg.* **148**(3), 220–234 (2022). 34967895 10.1001/jamaoto.2021.3767 PMC8719275

